# Biological and biomimetic materials and surfaces

**DOI:** 10.3762/bjnano.8.42

**Published:** 2017-02-08

**Authors:** Stanislav Gorb, Thomas Speck

**Affiliations:** 1 Department of Functional Morphology and Biomechanics, Zoological Institute of the University of Kiel, Am Botanischen Garten 9, 24118 Kiel, Germany; 2 Plant Biomechanics Group & Botanic Garden, Faculty of Biology, University of Freiburg, Schänzlestr. 1, 79104 Freiburg, Germany,; 3 Freiburg Institute for Interactive Materials & Bioinspired Technologies (FIT), 79104 Freiburg, Germany

**Keywords:** adhesion, bio-inspired materials, biomimetics, interfaces, lotus effect, surfaces

This Thematic Series is a tribute to Wilhem Barthlott, a famous German botanist, an observant naturalist, and a truly outstanding personality, on the occasion of his 70th birthday. One of his most important achievements was building a bridge between systematic studies of plant surfaces and the nano-/microtechnology of superhydrophobic, self-cleaning, and air-holding technical surfaces.

From everyday life experience, we all know that during watering or rainfall, water rolls off the leaves of many plants in the form of spherical droplets leaving the leaves themselves entirely dry. This effect can be seen in an especially impressive manner on the leaves of the sacred lotus (*Nelumbo nucifera*) and Tropaeolum (*Tropaeolum majus*). Interestingly, with the rolling off of water droplets, dirt particles as well as fungus spores and bacteria are also very efficiently removed from the leaf surfaces as they are more tightly attached to the water droplet than to the leaf surface. The reason for this physicochemical “self-cleaning behaviour” is the double-structured water-repellent surface of the plant leaves which is comprised of micro- and nanostructured wax on dome-shaped papillose epidermis cells. Dirt particles as well as microorganisms and fungus spores are attached only via a few contact points to the leaf surface which has a micro-/nanoscale roughness. The same holds for the water droplets which become spherical for energetic reasons and cannot wet the leaf surface [1–3].

The discovery of this phenomenon and its structural basis dates back to the 1970s when Wilhelm Barthlott studied the micro- and nanostructures on the surfaces of plant leaves, flowers, seeds and pollen during his Ph.D. thesis at the University of Heidelberg using one of the first scanning electron microscopes (SEMs) available for German botanists. His main interest in these years was plant systematics and he was interested if the fascinating nano- and microstructures he saw in the SEM are of importance for classifying the different plant taxa; this was proved true for many of these structures. Wilhelm Barthlott was especially interested in the tiny lipid structures, the so-called plant waxes, which later proved to be wax crystals that are formed by self-organisation processes and show specific shapes characteristic for different plant groups. To study these surface structures in the SEM, the leaves have to be prepared and cleaned, and Wilhelm Barthlott soon realized that the leaves of some plant species were always clean whereas the surfaces of other species were always dirty. Surprisingly the smooth, wettable leaf surfaces were markedly dirty, whereas the water-repellent, hydrophobic leaf surfaces with microscale roughness were always clean. More than 40 years ago he verified this observation by performing simple experiments with Tropaeolum leaves and published the hypothesis that, in a specific manner, double-structured, hydrophobic surfaces are self-cleaning. He further hypothesized that this may be the main biological function of the micro-/nanostructured wax covering many plant leaves [4]. This first publication was largely ignored outside of the botanical community and the importance of this observation was, at that time, not realized by any surface science physicists or chemists. It took about 15 years before in the 1990s Wilhelm Barthlott, now serving as chair for Botany at the University of Bonn, started new studies based on his earlier work together with his then student Christoph Neinhuis, who now has a chair for botany at the Technical University of Dresden. Together they detailed and quantified the structural and functional basis of the self-cleaning effect with numerous more sophisticated experiments. They also proved that in order to establish comparable self-cleaning properties on technical surfaces, the micro- and nanostructures found on the plant leaves and the hydrophobicity of the plant waxes must be transferred [1,5–6]. This has successfully been done in some biomimetic products, for example, the facade paint Lotusan^®^ produced by Sto SEA Pte. [7] or the product Tegotop^®^ 210 from Evonik Industries AG. The products are sold under the brand name Lotus-Effect^®^ which has become a near synonym for functional, water-repellent surfaces in general. Without exaggeration one can say that Lotus-Effect^®^ surfaces, together with fasteners inspired by gecko attachment structures, can be considered as “flagships” of contemporary surface-related biomimetic research. Still today questions related to these effects are the topic of novel state-of-the-art studies in the fields of basic and applied research, as can be seen in some articles of this Thematic Series.

A second type of surface structure that has been observed is the so-called *Salvinia* effect that has been quantitatively characterized in collaboration with Thomas Schimmel from the Karlsruhe Institute of Technology (KIT) [8]. The biomimetic potential of this effect was first understood in the early 2000s by Wilhelm Barthlott. The swimming ferns of the genus *Salvinia*, but also other swimming and diving organisms (e.g., some spider and bug species as well as a few birds and mammals), typically possess double-structured, flexible, hairy structures on their outer surfaces which can trap an air layer under water for time spans of several minutes, days and even months. The biological importance of these air layers may be buoyancy, insulation, oxygen supply during diving and/or friction reduction during swimming and diving. The main interest in biomimetic products lies in the potential friction reduction and in antifouling properties. For example, this could be practically implemented by covering the underwater parts of ship hulls with *Salvinia* effect coatings. Ongoing projects with ship builders aim to realize this biomimetic application [9].

In addition to the fields of botanical–biomimetic research, Wilhelm Barthlott has significantly contributed to many other fields of botany, for example, systematics and functional morphology of carnivorous plants and epiphytic cacti, biogeography/biodiversity and pollination biology (UV signatures). Some of these topics have become objects of interest in biomimetic research and are partially covered in articles in this Thematic Series [10–11].

For his studies on self-cleaning surfaces, and especially for the successful transfer of this technology to bioinspired self-cleaning technical products, Wilhelm Barthlott has been awarded with several prestigious prizes, for example, the Philipp-Morris Forschungspreis, the Karl Heinz Beckurts-Preis and the Deutscher Umweltpreis, to name just a few.

This Thematic Series of the *Beilstein Journal of Nanotechnology* is a collection of papers ranging from studies on structure–property relationships of biological materials and surfaces to the development of technical systems inspired by biological studies. In other words, this issue aimed at isolating the different stages of the biomimetic process. On one hand, it reflects the research phases of Wilhelm Barthlott and his collaborators that crossed from biological observations to the development of technical products. On the other hand, it shows the variety of facets of biomimetic research in general. This Thematic Series also covers a wide range of biological and technical systems and can be interesting for biologists, physicists, chemists, as well as materials scientists and engineers fascinated by biomimetics. The first article addresses the general relationship between universities, society, industry, and discuss borders within universities, borders in thinking, and the great amount of energy loss due to these borders. In the second part of this paper, Neinhuis demonstrates the impact of the research conducted by Wilhelm Barthlott and highlights the fact that, throughout his scientific career, many of the above-mentioned borders were removed, shifted or became more penetrable [12].

## Plant systems

Poppinga et al. conducted snap–trap closure experiments in air and under water and presented strong evidence that adult snaps of the Venus flytrap (*Dionaea muscipula*) are similarly fast in aerial and submersed states. The authors showed that minute seedling traps are much slower and do not yet incorporate elastic instabilities responsible for the fast motion. These findings are discussed in biomechanical and biomimetic contexts [10].

The paper by Masselter et al. is devoted to the mechanically relevant structures and their mechanical behaviour in various arborescent and shrubby monocotyledons plants with an emphasis on the structure–function relationships in *Dracaena marginata* stems [11]. Based on the results of microscopy and mechanical testing, a model of mechanical interactions between tissues and vascular bundles in the *D. marginata* stem was generated, and the potential significance of the results for the development of branched and unbranched bio-inspired fibre-reinforced systems with enhanced properties is discussed.

The hydrated mucilage of the *Plantago lanceolata* seed causes specific adhesive and frictional properties, playing an important role in seed dispersal. Kreitschitz et al. studied these tribological properties of the seed envelope under different hydration conditions and revealed the presence of cellulose fibrils in the mucilage in a microscopy study, which are presumably responsible for the uniform distribution of the mucilaginous layer on the seed surface. They may additionally protect the mucilage against the mechanical and chemical impact of the animal digestion systems [13].

## Animal systems

Resilin is a rubber-like protein derived from arthropod exoskeletons. It is composed of a stable network of randomly orientated peptide chains that are covalently cross-linked by dityrosine and trityrosine. Due to this molecular structure, resilin bears a high flexibility and resilience. The review by Michels et al. summarizes the presence of resilin and its functional significance in membrane and joint systems, jumping and catapulting systems, attachment and prey catching systems, reproductive, folding and feeding systems, as well as in traumatic reproductive systems [14].

The diets of marine zooplanktonic copepod crustaceans comprise a large proportion of the diatom taxa whose silicified shells exhibit extremely impressive mechanical stability. The ability of copepod species to efficiently break stable diatom structures is based on feeding tools with strongly specialized material architecture, chemical compositions and mechanical properties. The paper by Michels et al. [15] is the review of recent studies on copepod feeding tools. Their siliceous teeth consist of composite materials with silica-based cap-like structures situated on chitin-bearing cuticle sockets that are connected through flexible resilient areas containing resilin protein. This composite architecture contributes to the performance of the siliceous teeth in damaging diatomes and increases their resistance to mechanical damage.

The thin hind wings of diving beetles (Dytiscidae) are fragile and protected by their elytra (leathery forewings). In the resting beetle, the hind wings are folded over the abdomen; in flight, they are unfolded in order to provide aerodynamic forces. The paper by Sun et al. demonstrates that the unfolding process of the hind wing of *Cybister japonicus* is driven by the haemolymph motion in the wing veins. The hind wing extending process is simulated in a numerical model. This finding can assist the design of new, bioinspired, deployable systems [16].

The adhesive tongues of frogs are an efficient tool capable of capturing fast moving prey. It is plausible that the interaction between the tongue surface and the adhesive mucus coating is important for generating strong pull-off forces. The paper by Kleinteich and Gorb is a comparative study of tongue surfaces in nine frog species [17]. All examined species bear microscopical papillae, but different species have microstructure of different shape. The specific microstructure might presumably contribute to the particular adhesive performance of different frog species and may correlate with the prey spectra between the taxa studied. This study opens an interesting possibility of combining surface microstructures with adhesive fluids to enhance dynamical performance of the next generation of adhesives.

The majority of insects bear adhesive foot pads, which are used in locomotion on smooth vertical surfaces and ceilings. The functioning of the pads depends on various environmental factors. Heepe et al. showed that the attachment performance of the beetle *Coccinella septempunctata* depends on the relative humidity. The authors demonstrated that both low (15%) and high (99%) environmental humidity leads to a decrease of attachment forces generated by beetles [18]. The paper by England et al. systematically investigated beetle attachment ability on eight different surfaces having different structural and physico-chemical properties. The results show that chemical surface properties had no considerable effect on the beetle attachment. In contrast, the surface micro- and nanotopography strongly affected measured attachment forces [19].

Animal adhesive systems also perform well on the majority of rough surfaces, but not on all of them. Rough surfaces can change the real contact area and thereby diminish the effectiveness of the biological adhesive system. In the paper by Crawford et al. [20], the authors experimentally tested the effect of surface roughness on the adhesive ability of the tree frog *Litoria caerulea*. They demonstrated that at small scale roughness, frog pad adhesion may even surpass that measured on smooth substrates, whereas at large scale roughness, the adhesion was reduced. Presumably, on the latter substrates, the pads secrete an insufficient amount of fluid to fill the gaps between the asperities [20].

## Modelling and biomimetic systems

Antony et al. [21] present the sustainability assessment of the facade paint Lotusan^®^, which is a well-known biomimetic development based on the research of Wilhelm Barthlott. The authors used criteria from the Association of German Engineers (VDI) to verify whether the product can be defined as biomimetic. Using a systematic comparative product sustainability assessment (PROSA), the authors demonstrated that this cost-effective and resource-saving product is indeed biomimetic. It is also shown that Lotusan*^®^* has a low environmental impact [21].

The regular solution theory was applied by Akerboom et al. to study the advancing and receding contact angles of a liquid drop. The authors additionally applied a three-gradient model for a liquid/vapour system in contact with a complex surface geometry [22]. The authors concluded that the air entrapment is presumably not the main reason for the advancing contact angle variability. Since the contact line between fluid and uneven solid surface is pinned and curved, it induces curvature perpendicular to the plane, and the contact line does not move continuously but rather through a set of depinning transitions. Therefore, it is concluded that the full 3D structure of the surface, rather than any simplified parameter, determines the final observed contact angle.

Chemical analyses of insect tarsal fluid motivated Speidel et al. to prepare 12 biologically inspired, heterogeneous, synthetic emulsions. The microscopic structure was analysed and adhesive, frictional, and rheological properties were tested. The authors have clearly demonstrated that by varying their chemical composition, synthetic heterogeneous emulsions can be adjusted to have diverse consistencies and mimic certain rheological and tribological properties of natural tarsal insect adhesives [23].

In one of the articles of this Thematic Series, Egorov et al. proposed a relatively simple protocol for 3D printing of complex-shaped biocompatible structures based on sodium alginate and calcium phosphate for bone tissue engineering [24]. The analysis of 3D printed structures shows that they possess large interconnected porous systems and compressive strengths from 0.45 to 1.0 MPa, which demonstrates the rather strong potential of this approach for fabrication of biocompatible scaffolds.

In general, this Thematic Series discusses numerous experimental methods for the characterization of the mechanical properties of biological materials and surfaces at the micro- and nanoscale. The Thematic Series combines approaches from biology, physics, chemistry, materials science, and engineering and therefore represents an example of modern interdisciplinary science. Due to this latter reason, we hope that the contributions from this Thematic Series will be of interest to both engineers and physicists, who use inspirations from biology to design technical materials and systems, as well as to biologists, who apply physical and engineering approaches to understand how biological systems function.

The editors would like to thank all the authors for contributing their first-class work to this Thematic Series. We are also grateful to all referees for their constructive reports which facilitated the high quality of the manuscripts and also allowed us to publish in a timely manner. Finally, we thank the Editorial Team at the Beilstein-Institut for their continuous great support of biology- and biomimetics-related topics in this journal.

Stanislav Gorb and Thomas Speck

Kiel and Freiburg, December 2016
